# Band structure engineering via piezoelectric fields in strained anisotropic CdSe/CdS nanocrystals

**DOI:** 10.1038/ncomms8905

**Published:** 2015-07-29

**Authors:** Sotirios Christodoulou, Fernando Rajadell, Alberto Casu, Gianfranco Vaccaro, Joel Q. Grim, Alessandro Genovese, Liberato Manna, Juan I. Climente, Francesco Meinardi, Gabriele Rainò, Thilo Stöferle, Rainer F. Mahrt, Josep Planelles, Sergio Brovelli, Iwan Moreels

**Affiliations:** 1 Nanochemistry Department, Istituto Italiano di Tecnologia, via Morego 30, IT-16163 Genova, Italy; 2 Department of Physics, University of Genoa, via Dodecaneso 33, IT-16146 Genova, Italy; 3 Departament de Quimica Fisica i Analitica, Universitat Jaume I, ES-12080 Castellón, Spain; 4 Dipartimento di Scienza dei Materiali, Università degli Studi di Milano-Bicocca, via Cozzi 55, IT-20125 Milano, Italy; 5 IBM Research—Zurich, Säumerstrasse 4, CH-8803 Rüschlikon, Switzerland

## Abstract

Strain in colloidal heteronanocrystals with non-centrosymmetric lattices presents a unique opportunity for controlling optoelectronic properties and adds a new degree of freedom to existing wavefunction engineering and doping paradigms. We synthesized wurtzite CdSe nanorods embedded in a thick CdS shell, hereby exploiting the large lattice mismatch between the two domains to generate a compressive strain of the CdSe core and a strong piezoelectric potential along its *c*-axis. Efficient charge separation results in an indirect ground-state transition with a lifetime of several microseconds, almost one order of magnitude longer than any other CdSe/CdS nanocrystal. Higher excited states recombine radiatively in the nanosecond time range, due to increasingly overlapping excited-state orbitals. *k̇p* calculations confirm the importance of the anisotropic shape and crystal structure in the buildup of the piezoelectric potential. Strain engineering thus presents an efficient approach to highly tunable single- and multiexciton interactions, driven by a dedicated core/shell nanocrystal design.

## Abstract (web summary)

Quantum dots confine electrons to a nanometre length scale, and this gives rise to numerous quantum effects. Here, the authors directly control the excitonic structure of nanocrystal quantum dots by manipulating intra-particle piezoelectric fields.

## Graphical abstract

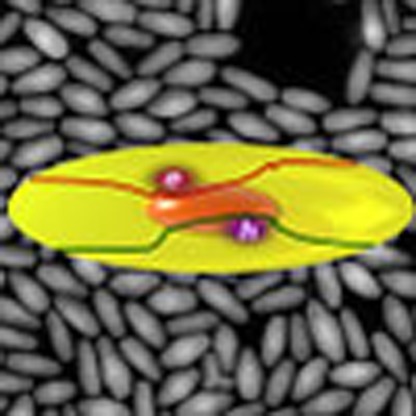

Gaining control over strain, through externally applied forces or via epitaxial growth of heterostructures with different lattice constants, is an important aspect of materials science. In particular, for optoelectronic and photonic applications, the addition of small distortions to the periodic lattice through inclusion of tensile or compressive strain shapes the band structure of semiconductors and the local band offset at the interface between different materials. Strain engineering has already found applications in diverse fields, with earlier examples showing, for instance, improved performance of silicon field-effect transistors via modified carrier mobilities^1^,^2^ and reduced lasing thresholds in strained InGaAs quantum wells^3^. Uniaxial strain is also predicted to induce an indirect-to-direct bandgap transition in germanium^4^, which could lead to efficient light emission from group-IV materials^5^. In epitaxial quantum dots, strain is used to switch between heavy and light holes in the ground-state exciton^6^, or to minimize the μeV fine-structure splitting between bright excitons^7^.

Colloidal nanocrystals (NCs) form a particular class of materials in which strain can be exploited to unprecedented levels. Due to the small dimensions of the template-free NCs (<100 nm), they can withstand significant elastic deformations^8^. In small NCs, the equilibrium lattice constant even differs from bulk, as demonstrated in ZnS (1% contraction)^9^ or PbSe (0.8% dilatation)^10^. Hence, colloidal core/shell heteronanocrystals (heteroNCs) can be grown under large lattice mismatch conditions, leading to for instance high-quality CdSe/CdS (4.4% mismatch for zincblende, ZB, crystal structure), CdTe/CdSe (6.4%) and CdTe/ZnSe (13.4%) NCs^11^,^12^,^13^. Furthermore, in several systems local band warping at the core/shell interface has been observed^14^,^15^,^16^. In CdTe/ZnSe heteroNCs, strain has been employed to invert the band offset from a type-I to a type-II heterostructure^11^, and for CdSe/CdTe it has a strong influence on the carrier relaxation and recombination dynamics^17^,^18^,^19^,^20^. Nevertheless, in most applications interfacial strain is avoided, since it may also lead to the formation of interface defects that reduce the emission efficiency^12^,^13^.

Strain engineering can also be a means to construct a unique type of nanostructure, typically in NCs with a non-centrosymmetric crystal lattice. The absence of an inversion centre leads to a range of symmetry-dependent properties such as piezo- and pyroelectricity, chirality and circular dichroism^21^. Strain-induced piezoelectric fields have been demonstrated to lead to a significantly modified band structure and electron–hole overlap in CdSe/CdS Stark superlattices^22^ and epitaxial III-nitride quantum dots^23^. This effect resulted from the wurtzite (WZ) crystal structure and from lattice mismatches of 2.5 and 4.2%, and 4 and 3.9% (ref. 24), along *a*- and *c*-axes, respectively, that give rise to piezoelectric fields larger than 10^6^ V cm^−1^, an order of magnitude stronger than in ZB crystals^22^,^25^. In colloidal NCs, it would imply a new direction in controlling the optoelectronic properties, in addition to quantum confinement via the NC size and shape, band structure engineering through the growth of heterostructures^11^,^12^,^13^ or controlled doping to induce highly localized electronic states^26^.

In this work, we demonstrate a direct control over the excitonic properties of NC quantum dots, through creation and manipulation of strong intra-particle piezoelectric fields, driven by the engineered interfacial strain in a new class of colloidal heterostructures. Using WZ rod-in-rod (RIR) CdSe/CdS NCs with minimum dimensions larger than the respective bulk Bohr radii of CdSe and CdS, 5.6 and 2.8 nm, respectively, we exploit the strain-induced piezoelectric fields to create two spatially separated potential wells for electrons and holes at opposite sides of a 26 nm long CdSe nanorod. We hereby constructed a long-lived, indirect exciton with a lifetime of 4.4 μs, which is almost an order of magnitude longer than decay times previously reported for CdSe/CdS heteroNCs^27^,^28^,^29^. In the multiexciton regime, such a band structure implies nearly unscreened repulsive Coulomb interactions between electrons and holes. Consequently, excited-state carriers still easily escape the shallow potential wells, leading to a large electron–hole wavefunction overlap for higher excited states with efficient, blue-shifted multiexciton emission and nanosecond recombination dynamics. Next to the unique approach to using internal electric fields to design the carrier potential wells, the relatively large dimensions, with strong electron–hole separation and associated microsecond exciton lifetimes, multiexciton blue shifts and reduced Auger recombination, give rise to a nanostructure that is optimally shaped for photonic applications ranging from gated fluorescence and exciton storage^30^ to single-exciton lasing^31^, via an exceptional degree of control over the charge carrier localization.

## Results

### RIR strain characterization

**Figure 1:**
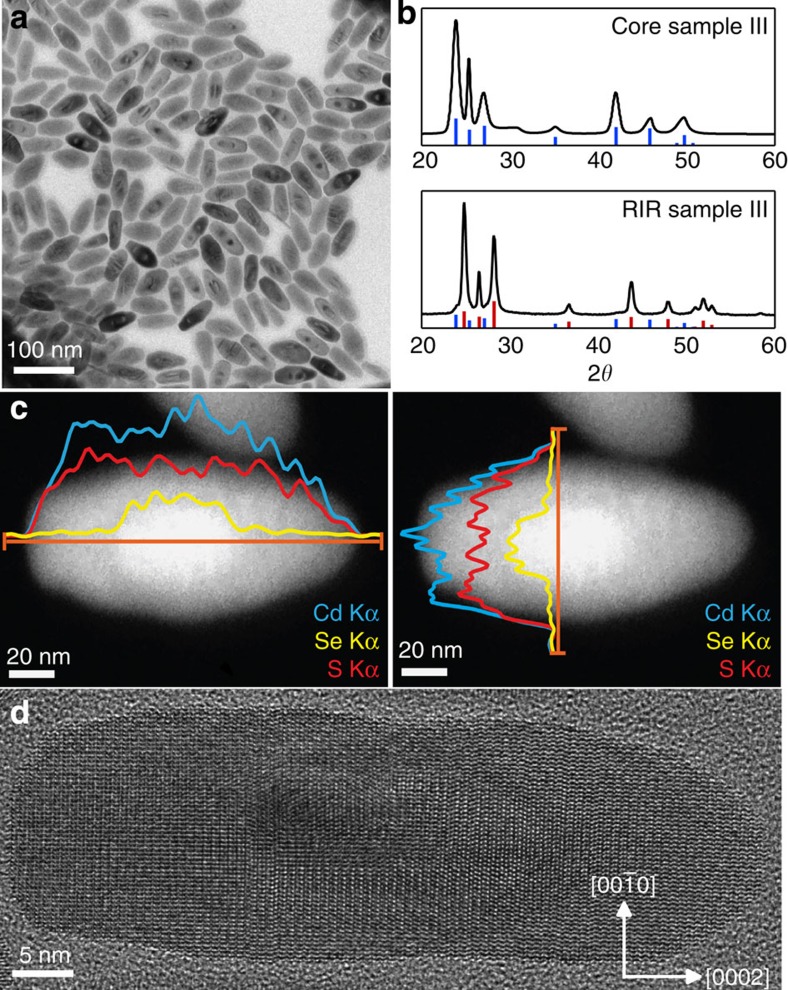
Structural characterization of the RIRs. (**a**) Overview TEM of CdSe/CdS RIR sample III. The dark and light areas around the CdSe cores are due to diffraction contrast generated by the local distortion of the lattice near the core/shell interface. (**b**) XRD patterns of core CdSe nanorods and corresponding core/shell RIRs (sample III). Vertical lines indicate the bulk WZ CdSe (blue) and CdS (red) diffraction angles. (**c**) HAADF-STEM image of single RIRs, with projected composition determined from the EDS profile (along orange line) of Cd Kα, S Kα and Se Kα signals. (**d**) HRTEM image of a single RIR (Sample III) showing epitaxial growth of CdSe/CdS core/shell heterostructures.

The synthesis of CdSe nanorods is adapted from the procedure described by Miszta *et al*.^32^, and yields NCs with sizes from 3 nm (diameter) by 9 nm (length), up to 11 nm by 26 nm, that is, beyond the strong confinement regime (Table 1; Fig. 1a; Supplementary Fig. 1). To obtain a sizable CdS shell around such large CdSe cores, we introduced CdCl_2_ in the synthesis^32^,^33^, which activates the shell growth via otherwise unreactive facets in accordance with the synthesis of CdSe/CdS octapods^33^. This avoids homogeneous nucleation of CdS clusters and leads to an overall 30 nm by 85 nm heteroNC in typically 10 min (Table 1; Supplementary Figs 1 and 2). Structural characterization by X-ray diffractometry (XRD) confirms the growth of WZ core/shell RIRs (Fig. 1b). XRD patterns of both CdSe seeds and CdSe/CdS heteroNCs display sharper [0002] diffraction peaks around 25° 2*θ*, confirming that the *c*-axis corresponds to the NC long axis. As evidenced by high-angle annular dark-field scanning transmission electron microscopy (HAADF-STEM) and elemental analysis with energy-dispersive X-ray spectroscopy (EDS) line scans (Fig. 1c), the growth in both directions along the *c*-axis also yields more symmetric core/shell NCs, with the CdSe core nanorod located at the centre of the heterostructure. This is in contrast with the seeded growth of CdSe/CdS dot-in-rods (DIRs)^34^, and previous RIR syntheses^35^, where the core is typically located towards one end of the heteroNC. High-resolution TEM (HRTEM) shows few stacking faults within the heteroNCs and confirms an epitaxial shell growth according to the following symmetry relationships: CdS[0002]//CdSe[0002] and CdS
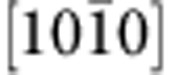
//CdSe
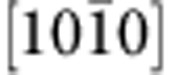
 (Fig. 1d; Supplementary Fig. 3; Supplementary Note 1). Raman spectroscopy revealed that, despite the fast growth of the shell, the RIR NCs exhibit a CdSe_*x*_S_1−*x*_ alloy region at the CdSe/CdS interface (Supplementary Fig. 4; Supplementary Table 1; Supplementary Note 2), likely due to the 380 °C growth temperature used.

**Figure 2:**
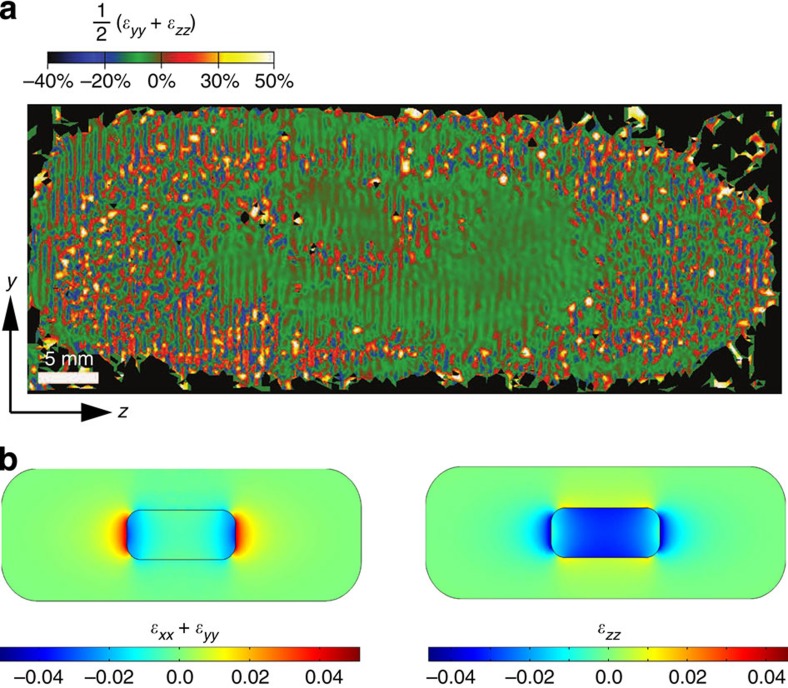
Experimental and theoretical strain analysis. (**a**) Mean dilation strain map of the RIR NC shown in Fig. 1c (scale ranges from −40% to 50% for an optimum contrast). Few stacking faults and dislocations locally distort the strain field, yet we observe a region of homogeneous strain (green) of about 15 × 30 nm, clearly larger than the 11 × 26 nm core CdSe nanorod. The *z*- and *y*- tensor axes correspond to [0002]** and 
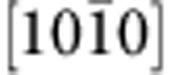
 crystallographic axes, respectively. (**b**) Calculated lateral (left panel) and *c*-axis (right panel) strain tensor components, showing the compression of the CdSe core and dilatation of the CdS shell at the CdSe/CdS interface.

Several RIR NCs in the bright-field TEM image (Fig. 1a) show a particular contrast, occurring either near the tips of the CdSe core or along its sides. This can be attributed to a local deformation of the crystal lattice near the CdSe/CdS interface (Ashby–Brown diffraction contrast^36^,^37^,^38^), and already gives a first indication of significant strain in our NCs. To further evaluate the deformation, we calculated the strain fields of the HRTEM images (Fig. 2d) using the peak pairs analysis^39^. The coordinates of the atomic *u*_*ij*_ displacements were chosen such that the *z*-axis of the strain maps was parallel to the crystallographic *c*-axis, and the *y* axis corresponded to the 
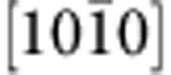
 axis. Figure 2a shows the resulting mean dilatation strain map extrapolated from the HRTEM image reported in Fig. 1c. It reveals a significant dilatation of the CdS lattice near the CdSe core (central green region), with respect to the CdS crystal lattice at the far ends of the heteroNC. Importantly, due to the central position of the CdSe core nanorod and the epitaxial growth of the CdS shell, the lattice deformation can develop symmetrically at both sides of the heterostructure. We quantified the (projected) area with a uniform crystal lattice (Supplementary Table 2), and found that the diameter obtained via HAADF-STEM and EDS line scans is 1.4 nm smaller than the strain profile. A more pronounced extension of the core structure is observed in the axial direction, by 5.0 nm on either side. This can be ascribed to a significant lattice deformation inside the CdSe core and at the core/shell interface, which extends the strain field into the CdS shell.

Experimental data are supported by theoretical calculations of the strain. Figure 2b shows the lateral (*ɛ*_*xx*_+*ɛ*_*yy*_) and axial (*ɛ*_*zz*_) strain tensor components of a large RIR, defined with respect to the natural lattice constants of the CdSe core and the CdS shell in each region. The strong compressive strain of the core along the *c*-axis (*ɛ*_*zz*_<0), along with the tensile strain of the shell surrounding the lateral sides (*ɛ*_*zz*_>0), show that both materials tend to minimize the structural mismatch on the lateral interface by deforming the axial lattice constants. The same occurs with the lateral strain near the CdSe core tips, where the core is again compressed (*ɛ*_*xx*_+*ɛ*_*yy*_<0) and the shell is dilated (*ɛ*_*xx*_+*ɛ*_*yy*_>0). The extent of compression and dilatation is in line with the expansion of the core crystal lattice into the shell, as observed by HRTEM strain analysis. More importantly, the elastic behaviour of the RIR lattice implies that the compression/dilatation of core and shell is followed by an opposite response in the orthogonal directions. This leads to a very weak lateral strain on the long sides of the rod (core and shell roughly preserve their natural in-plane constants) and a strongly compressive axial strain of the CdS lattice near the tips. The severe anisotropy of the strain in these structures gives rise to strong polarization along the axial direction, which translates into the intense piezoelectric field. The large thickness of the CdS shell leads to a situation where the outer CdS monolayers are nearly undeformed. As a result, slightly different shell thickness within this size regime should have no significant influence on the strain^40^.

### Band structure and optical properties

**Figure 3:**
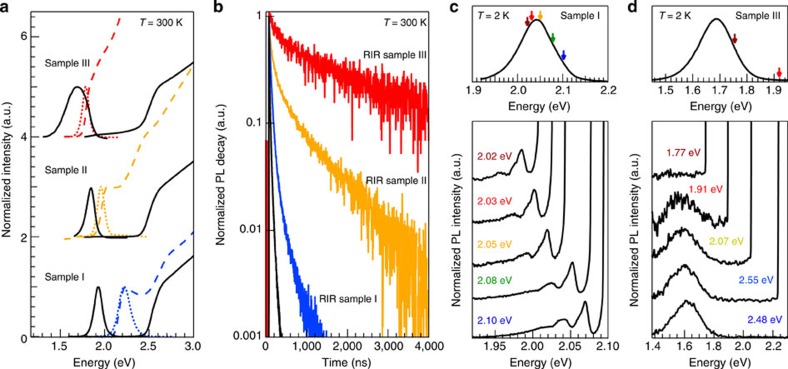
Optical confirmation of the indirect exciton formation. (**a**) Room temperature absorbance spectra of CdSe core nanorods (dashed lines) and CdSe/CdS core/shell RIRs (full lines), with corresponding PL spectra of core (dotted lines) and core/shell RIRs (full lines). Note that in RIR sample III, Rayleigh scattering is observed, possibly due to the large RIR volume. (**b**) Time-resolved PL decay traces of CdSe nanorods (black), and RIR samples I, II and III. We observe a lifetime up to 4.4 μs, for RIR sample III. (**c**,**d**) Fluorescence line-narrowing (FLN) spectra at 2 K of quasi-type-II heteroNCs (RIR sample I, **c**) and type-II heteroNCs (RIR sample III, **d**). The full PL spectrum under non-resonant excitation at 3.1 eV is plotted on top for reference, with arrows indicating the excitation wavelength.

With experimental and theoretical verification of the presence of the strain in our WZ RIR heteroNCs, we proceeded with measuring its influence on the single-exciton and multiexciton emission properties. In Fig. 3, we report the optical absorption and photoluminescence (PL) spectra of three representative CdSe/CdS RIRs with increasing core size (PL excitation spectra are reported in Supplementary Fig. 5). Interestingly, upon growing the CdS shell, we observed a significant red shift of the PL spectrum even for the largest RIRs (RIR sample III, Fig. 3a), whose PL peaked at 1.69 eV. This emission energy is well below the bandgap energy of bulk CdSe (*E*_g_=1.74 eV) and thus provides a first indication of an indirect exciton transition. Time-resolved PL decay traces provide further evidence of a significantly reduced electron–hole overlap in strain-engineered RIR NCs with respect to conventional unstrained materials (Fig. 3b). Remarkably, we obtained PL lifetimes varying from *τ*=100 ns, for the RIR with the smallest core (RIR sample I), up to *τ*=4.4 μs for the largest RIR (RIR sample III). This value is over two orders of magnitude longer than the emission lifetime of the corresponding core-only CdSe nanorods (15–20 ns, Table 1), and surpasses also any precedent literature data on CdSe/CdS giant-shell DIDs with ZB^28^,^29^ and WZ cores^27^, which exhibit lifetimes up to 650 ns, and even intrinsic type-II CdSe/CdTe quantum dots^41^, nanorods^42^ and two-dimensional heteroplates, with lifetimes reaching 1.8 μs^43^. Note that the long lifetime here is not due to trap state emission, as we observed a continuous PL shift and increase in PL lifetime upon shell growth, in line with a progressive reduction of the electron–hole overlap (Supplementary Fig. 6). In addition, similar to spherical giant-shell NCs^12^,^13^,^27^,^28^,^44^, due to the thick CdS shell the electron and hole wavefunctions are efficiently decoupled from CdS surface traps, leading to a consistently high PL quantum efficiency of 15, 10 and 23% for the samples I, II and III, respectively.

To further confirm the transition from direct to indirect ground-state excitons in strain-engineered RIRs, we performed fluorescence line-narrowing (FLN) experiments at 2 K. Here, samples are excited by a narrow-band continuous-wave light source tuned to the red side of the band-edge absorption, which allows photoexciting a small fraction of the NC ensemble. As a result, we can resolve the spectroscopic features of longitudinal optical (LO) phonon-assisted transitions^45^,^46^. Resonant excitation of RIR sample I yields a structured FLN spectrum showing the typical vibronic progression associated with LO phonon modes of CdSe (LO_CdSe_=25 meV) and CdS (LO_CdS_=35 meV), and respective overtones (Fig. 3c; Supplementary Fig. 7). This indicates a so-called quasi-type-II band offset, characterized by a partial delocalization of the electron wavefunction over both the CdSe core and the CdS shell. On the other hand, resonant excitation of RIR sample III at 1.77 eV yields no measurable emission even for very long accumulation times (as long as 1 h). A weak PL is first observed by exciting the RIRs at least 140 meV above the energy of the PL maximum at 1.9 eV, at which point a featureless PL spectrum appears due to the excitation of the entire RIR ensemble (Fig. 3d). This clearly demonstrates that strained RIRs possess a large intrinsic Stokes shift that, together with the microsecond-long PL lifetime, is an unambiguous signature of indirect exciton formation.

**Figure 4:**
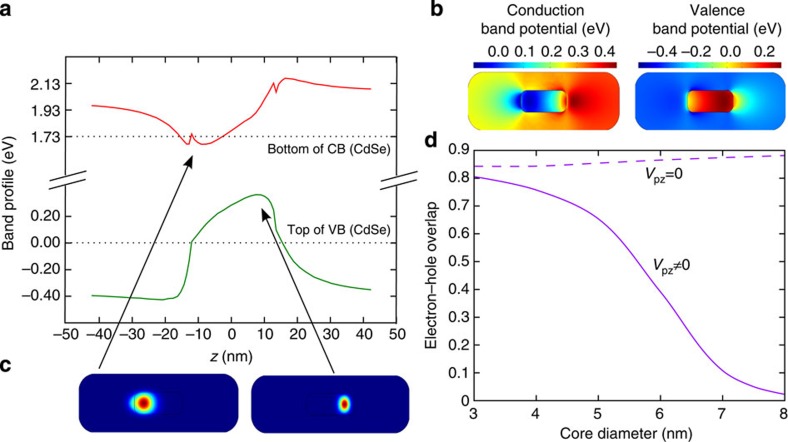
*k·p* calculations of the CdSe/CdS band structure. (**a**) Plot of the overall band structure along the long axis of the RIR sample III. The potential wells are strongly asymmetric, with a valence band maximum and conduction band minimum at opposite sides of the core CdSe nanorod. The indirect transition also has an energy below the bulk CdSe bandgap. (**b**) Corresponding two-dimensional plots. (**c**) Electron (left) and hole (right) wavefunctions are well separated, confirming the indirect exciton ground state. (**d**) The electron–hole overlap for RIRs of increasing size rapidly decreases when piezoelectricity is taken into account (solid line), while it otherwise remains above 80% (dashed line). The core has an aspect ratio of 1:3 and the shell is twice as large.

The observed optical properties of RIRs are markedly different from those of CdSe/CdS heteroNCs with a spherical core, where a transition to type-II behaviour typically occurs only in the limit of strong confinement and core diameters below 3 nm^47^,^48^. This strongly suggests that the strain must have a profound influence on the electronic structure of our elongated heteroNCs that have considerably bigger core diameters. Indeed, *k·p* calculations show that the hydrostatic strain potential already raises the CdSe conduction band, but a type-I band offset is still preserved (Supplementary Fig. 8). What truly differentiates the RIRs from previously reported DIDs and DIRs is the strain-induced piezoelectricity in combination with the elongated shape of the WZ CdSe core. In the largest RIR, the 0.15 MV cm^−1^ piezoelectric field builds up a potential difference of 0.45 eV across the long axis of the CdSe core (Fig. 4a,b; Supplementary Figs 9 and 10). This yields a local conduction band minimum and valence band maximum at opposite sides of the CdSe core nanorod, with electrons and holes that are almost completely separated and confined to their individual, 0.22 eV shallow potential wells (Fig. 4c). Considering an initial conduction band offset of 0.32 eV (ref. 49), we estimated a reduction of the electron–hole wavefunction overlap by two orders of magnitude after shell growth, consistent with the strong increase in lifetime observed experimentally (4.4 μs for RIR sample III, compared with 24 ns observed for the corresponding core-only CdSe nanorods). The overlap can be controlled by the rod length, eventually returning to a reduced piezoelectric field and more symmetric band structure in RIR sample I, where the electron–hole overlap is again mostly dominated by the band offset at the CdSe/CdS interface (Supplementary Fig. 11). The critical role of piezoelectricity in determining the electron–hole overlap of single excitons in RIR is further evidenced in Fig. 4d, which compares the calculated overlap for a series of RIRs with increasing size (aspect ratio: 1:3). A pronounced damping of the electron–hole overlap takes place when piezoelectricity is considered (solid line), which is otherwise missing (dashed line). Similar results are obtained with different conduction band offsets (Supplementary Table 3), which indicates that the role of piezoelectricity is robust within the uncertainty range of model parameters. It clearly shows that the piezoelectric effect induces carrier (de-)localization beyond what can be reached with quantum confinement or interfacial hydrostatic strain alone. These heteroNCs should therefore also be highly sensitive to external pressure or voltage, providing an interesting prospect as stress gauge^50^ or charge sensor^51^.

### Multiexciton emission

**Figure 5:**
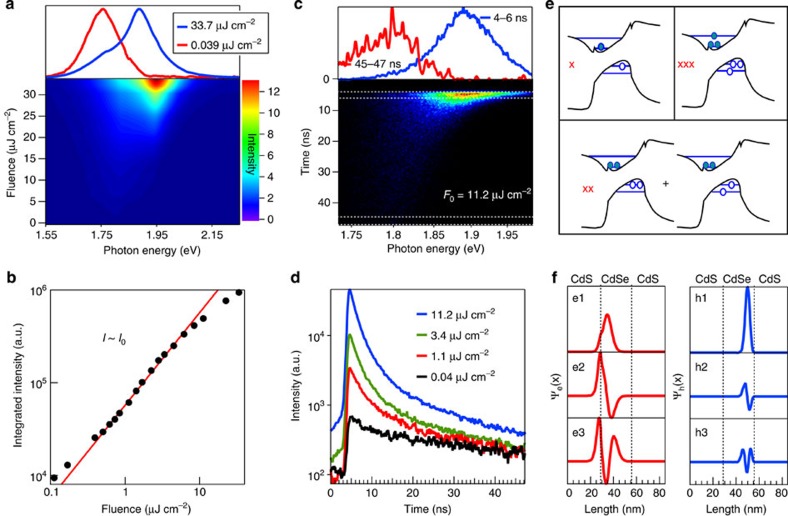
Multiexciton emission in RIRs. (**a**) Fluence-dependent PL spectra of RIR sample III. At high fluence, a 140 meV blue shift of the emission is observed. (**b**) The PL intensity increases linearly with fluence up to 10 μJ cm^−2^, suggesting strongly suppressed Auger recombination. (**c**) Streak camera image at a fluence of 11.2 μJ cm^−2^, confirming the blue-shifted MX emission. The emission at 45 ns is shown for comparison; however, it does not yet correspond to the pure single-exciton PL, which should peak at 1.75 eV. (**d**) The spectrally integrated decay is multiexpontential, with lifetimes of 1.9 and 20 ns, respectively. Note that MX emission can already be discerned below 1 μJ cm^−2^, while the linear increase of the PL persists up to 10 μJ cm^−2^ (see **b**). (**e**) Sketch of the main electronic configurations of exciton, biexciton and triexciton ground states. The occupation of higher lying orbitals, whose piezoelectric confinement is weaker, enables stronger electron–hole overlap for bi- and triexcitons. (**f**) Electron and hole wavefunctions for the three lowest electron and hole orbitals in RIR sample III, showing an increasing shift towards the core centre.

In the light of the remarkable effects of strain on the single-exciton behaviour, we also expect substantial differences between the multiexciton (MX) features of strain-engineered RIRs with respect to conventional, non-strained materials. We prepared thin films of the RIRs and cooled the sample to 4 K (Supplementary Fig. 12) to measure the fluence-dependent emission spectra. The PL spectrum of RIR sample I displays a weak, 28 meV blue-shifted peak at high excitation fluence (Supplementary Fig. 13), consistent with biexciton emission observed on DIRs^48^ with a small CdSe core. RIR sample III on the other hand exhibits a far larger, 140 meV blue-shifted peak that clearly dominates the emission spectrum at high fluence (Fig. 5a). The linear growth of the PL intensity with increasing fluence (Fig. 5b) implies a strongly reduced Auger recombination in these heteroNCs, similar to giant-shell DIDs^46^, and is possibly efficiently suppressed by the elongated CdSe core^52^ and smoothed wavefunctions due to interface alloying^53^,^54^,^55^ (Supplementary Fig. 4). The emission dynamics at high fluence is multiexponential with nanosecond lifetime components of 1.9 and 20 ns, respectively (Fig. 5c,d), significantly longer than what is expected from volume scaling alone (300–400 ps^46^, taking a volume of 2,345 nm^3^ corresponding to the CdSe core). Considering the suppressed Auger recombination, the MX recombination must therefore involve electrons and holes with a significantly larger wavefunction overlap with respect to single-exciton carriers. *k·p* calculations support this view. Unique to this system, the shallow nature of piezoelectric potentials (Fig. 4a) yields excited-state orbitals that are increasingly delocalized over the CdSe core (Fig. 5e,f). Since the electronic configuration of multiexcitons is constructed from such orbitals, their electron–hole overlap will be increasingly large. In contrast to the indirect ground-state transition, we can therefore expect a substantially enhanced multiexciton radiative rate. Considering both the 140 meV MX blue shift and the three orders or magnitude difference between X and MX lifetimes, the results thus suggest that strain-engineered colloidal NCs open up new pathways towards single-exciton lasers^31^, due to the well-separated single-exciton emission, or even novel biexciton lasing concepts that suppress Auger recombination by shape-control^56^.

## Discussion

With large WZ CdSe rods embedded in giant CdS shells, we have synthesized a unique colloidal system with a band structure determined by piezoelectric fields. The resulting indirect excitons have lifetimes up to 4.4 μs, with an electron–hole delocalization that can be carefully controlled by the material parameters. Next to a respectable PL quantum efficiency of 10–23% (considering the type-II configuration), these materials also exhibit efficient multiexciton emission and suppressed Auger recombination. This may not only lead to deepened insights into the excited-state carrier dynamics in colloidal nanocrystals, but may also open the way to further improvements in quantum dot-based energy harvesting^57^,^58^,^59^ or optoelectronic^31^,^60^ applications. In addition, the exceptionally long lifetimes could find, among others, application in dye-sensitized solar cells^61^ or exciton storage devices^30^.

## Methods

### Materials

Tri-*n*-octylphosphine oxide (TOPO, 99%), tri-*n*-octylphosphine (TOP, 97%) and selenium (Se, 99.99%) were purchased from Strem Chemicals. Cadmium oxide (CdO, 99.99%), cadmium chloride (CdCl_2_, 99.99%), sulfur (S, 99.98%), ethanol, toluene and chloroform were purchased from Sigma-Aldrich. *N*-octadecylphosphonic acid (ODPA) and *n*-hexylphosphonic acid (HPA) were purchased from Polycarbon Industries.

### Synthesis of CdSe cores

CdSe seeds were synthesized using a procedure adapted from Miszta *et al*.^32^ For example, to obtain the largest nanorods with a diameter of 10.6 nm and length of 25.6 nm (core of RIR sample III), 3 g of TOPO, 50 mg of CdO, 80 mg of HPA and 260 mg of ODPA have been added into a flask and degassed for 1 h at 150 °C. Then the temperature was increased to 380 °C under an argon flow, and 2.6 ml of TOP was injected. When the temperature of 380 °C was re-established, 1 ml of a 0.15 M TOPSe solution was injected and the nanorods were grown for 10 min. To synthesize smaller nanorods, the TOPSe concentration, the precursor and ligand concentrations and reaction temperature and time were changed (Supplementary Note 3).

### Synthesis of CdSe/CdS RIRs

In a 50 ml round-bottom flask, 3 g of TOPO, 50 mg of CdO, 6 mg of CdCl_2,_ 80 mg of HPA and 260 mg of ODPA were degassed at 150 °C under vacuum for 1 h^32^. Then, under argon flow the temperature was further raised to 380 °C, and 2.6 ml of TOP was injected. After the temperature of 380 °C was re-established, a mixture of 0.5 g of TOPS (from a preheated, supersatured TOPS stock solution containing 96 mg of S in 1 ml of TOP) and 150 μl of a 3 μΜ solution of CdSe nanorods in TOP were added. The CdS shell was grown for 10 min. Next, the solution was rapidly cooled to room temperature and 10 ml of toluene was added. The RIRs were purified by adding 5 ml of ethanol and centrifuging them at 3,000 r.p.m. for 5 min, after which they were redispersed in chloroform. This was repeated three times, and the RIRs were finally dispersed in 5 ml of chloroform.

### Structural characterization

X-ray diffractograms were measured with a Rigaku SmartLab 9 kW diffractometer operated at 40 kV. The XRD samples were obtained by dropcasting the NCs onto a miscut silicon substrate. Bright-field TEM measurements were carried out on a JEOL-1100 TEM operating at an acceleration voltage of 100 kV. HRTEM was performed with a JEOL JEM-2200FS microscope equipped with a field emission gun working at an accelerating voltage of 200 kV, a CEOS spherical aberration corrector of the objective lens, which enables a spatial resolution of 0.9 Å, and an in-column Omega filter. HAADF-STEM images were collected using a spot size of 0.7 nm. The chemical composition of the RIRs was determined by EDS line scan analysis performed in HAADF-STEM mode, using a Bruker Quantax 400 system with a 60 mm^2^ XFlash 6T silicon drift detector according to the Cliff–Lorimer method. HRTEM images were also used for strain analysis with the peak pairs analysis method^39^. The structural displacements *u*_*ij*_ of the atomic columns were used to calculate the (projected) *yz* strain tensor components, 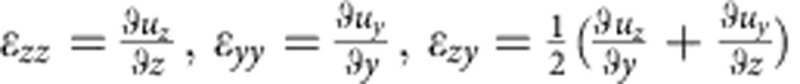, considering as the lattice basis vectors the [0002] and 
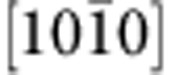
 structural reflections of the WZ lattice. The mean dilatation map was subsequently calculated from the mean value of the in-plane tensor 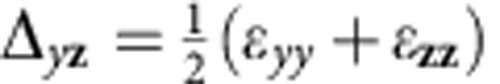.

### Raman spectroscopy

The NCs were dropcast on a glass substrate forming a close-packed film. Raman spectra were collected using a Renishaw InVia MicroRaman spectrometer, exciting the samples with a 100 mW diode laser at *λ*=532 nm, using a × 50 magnification microscope objective, with integration times up to 30 s.

### Single-exciton PL measurements

PL measurements were performed using an Edinburgh Instruments FLS920 spectrofluorometer. Samples were excited at 400 nm with a Xenon lamp for the steady-state measurements, and at 405 nm with a pulsed laser (50 ps pulse duration) for the time-resolved traces. Typically, a region of 10 nm around the peak maximum was selected for the decay measurements. Quantum efficiencies were determined with an integrating sphere, exciting the RIR sample I and II at 400 nm, and sample III at 450 nm. The optical density was tuned to 0.1 at the excitation wavelength.

### Fluorescence line-narrowing spectroscopy

For FLN measurements, a spectrally narrow photoexcitation source (<0.2 nm full width at half maximum) was produced by filtering the output of a 150 W Xenon lamp with a 1/3 m double-grating monochromator. The emitted PL photons were collected, fibre-coupled into a 1/2 m spectrograph and detected with a nitrogen-cooled charged-coupled device. All measurements were performed at 2 K.

### Multiexciton PL spectroscopy

RIR films were deposited on sapphire substrates and cooled to 4 K in a closed-cycle helium cryostat (Advanced Research Systems). To excite the samples, a 70 fs, 1 kHz Ti:sapphire amplified laser was frequency doubled to an excitation wavelength of 405 nm and reduced to a 2 mm (diameter) excitation spot. The PL was relayed to an Avantes spectrophotometer for steady-state measurements, and to a Hamamatsu Photonics streak camera to measure the time-resolved spectral decay.

### *k·p* Theory

Experimental data were compared with theoretical strain maps that were calculated in the continuous medium model by minimizing the elastic energy. The boundary conditions are zero normal stress for the free surface. Details on the underlying theory are given by Rajadell *et al*.^40^ The strain-induced piezoelectric polarization, which is linearly related to strain, is calculated by 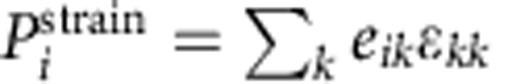. The determination of *ɛ*_*ij*_(***r***) is performed using the multiphysics mode of Comsol 4.2 software. Electrons (*j=e*) and heavy-hole (*j=hh*) states of the ground-state exciton are calculated from the Hamiltonian 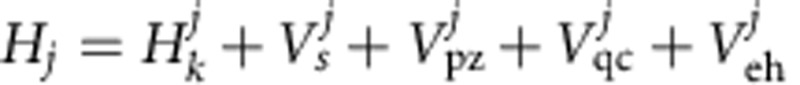, where 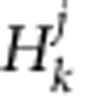 and 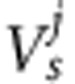 are the single-band effective mass kinetic energy and strain potential, 
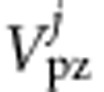
 is the piezoelectric potential, 
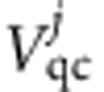
 the quantum confinement due to the different band offset of core, shell and surrounding environment materials and 
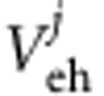
 the electrostatic potential created by the opposite sign carrier, which takes into account the dielectric mismatch with the dielectric surroundings of the RIR. Converged interacting electron and hole states are obtained by iterative resolution of the Schrödinger–Poisson equation (see Supplementary Table 4 for the material parameters).

## Additional information

**How to cite this article**: Christodoulou, S. *et al*. Band structure engineering via piezoelectric fields in strained anisotropic CdSe/CdS nanocrystals. *Nat. Commun.* 6:7905 doi: 10.1038/ncomms8905 (2015).

## Supplementary Material

Supplementary Information Supplementary Figures 1-13, Supplementary Tables 1-4, Supplementary Notes 1-3 and Supplementary References

## Figures and Tables

**Table 1:** Structural and optical properties of the different CdSe core nanorods and corresponding CdSe/CdS RIR samples.

**Sample**	**Diameter (nm)**	**Length (nm)**	**Emission (nm)**	**Lifetime (ns)**
Core sample I	3.2	8.9	560	14
RIR sample I	9.7	52.0	643	102
Core sample II	4.8	15.0	633	15
RIR sample II	9.8	43.8	670	853
Core sample III	10.8	25.6	695	24
RIR sample III	28.9	84.8	733	4,450

RIR, rod in rod.
