# Supplementary material for: Band structure engineering via piezoelectric fields in strained anisotropic CdSe/CdS nanocrystals
Source: Nat Commun. 2015 Jul 29;6:7905. doi: 10.1038/ncomms8905 (PMC4532876; doi:10.1038/ncomms8905)
Supplement: Supplementary Information — Supplementary Figures 1-13, Supplementary Tables 1-4, Supplementary Notes 1-3 and Supplementary References [file ncomms8905-s1.pdf]

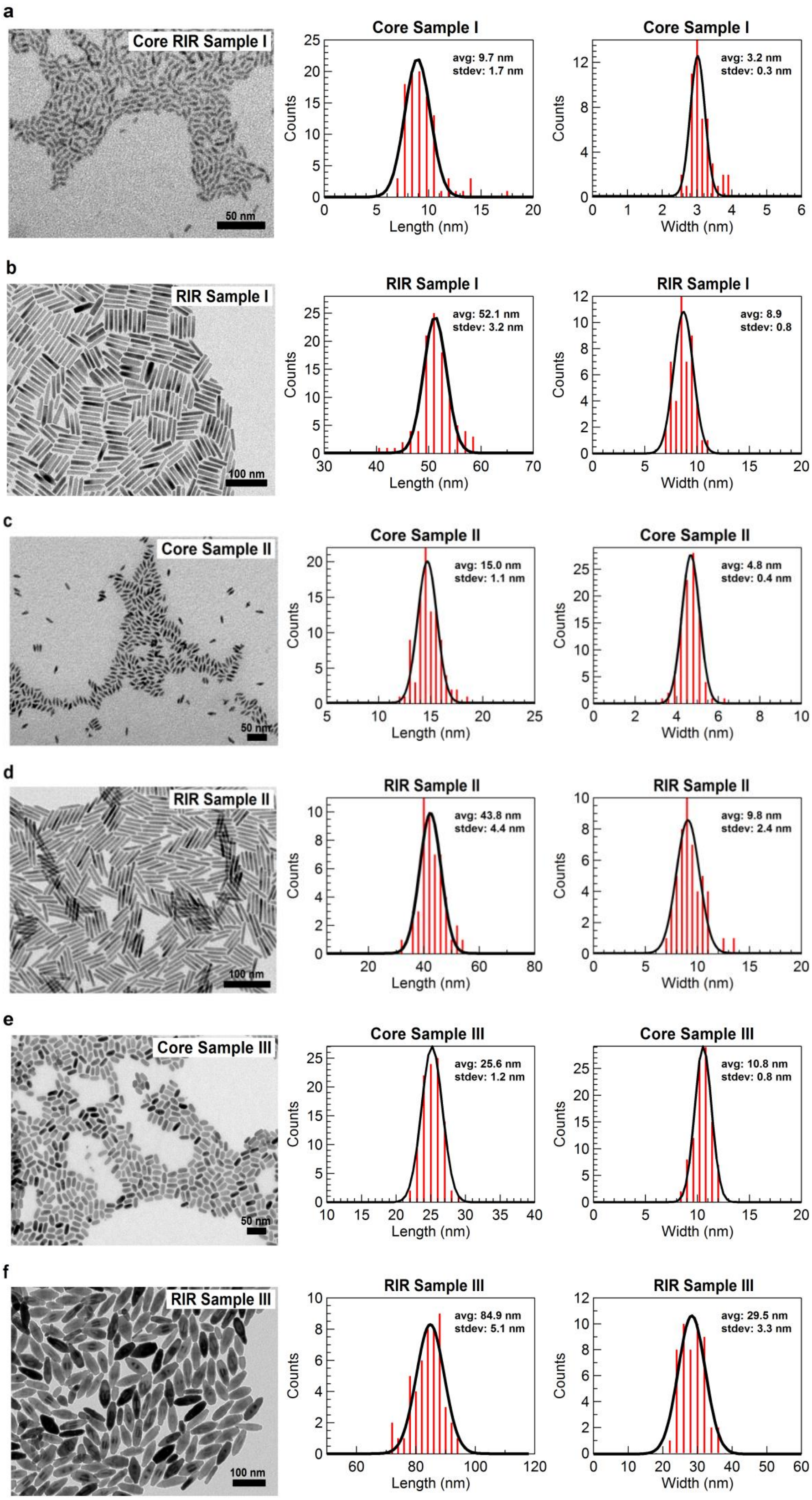

Supplementary Figure 1. Overview TEM images and corresponding histograms of CdSe core and CdSe/CdS core/shell NCs. The length and diameter of the different CdSe and CdSe/CdS NCs were determined from a Gaussian fit to the length and width distribution of 50 NCs.

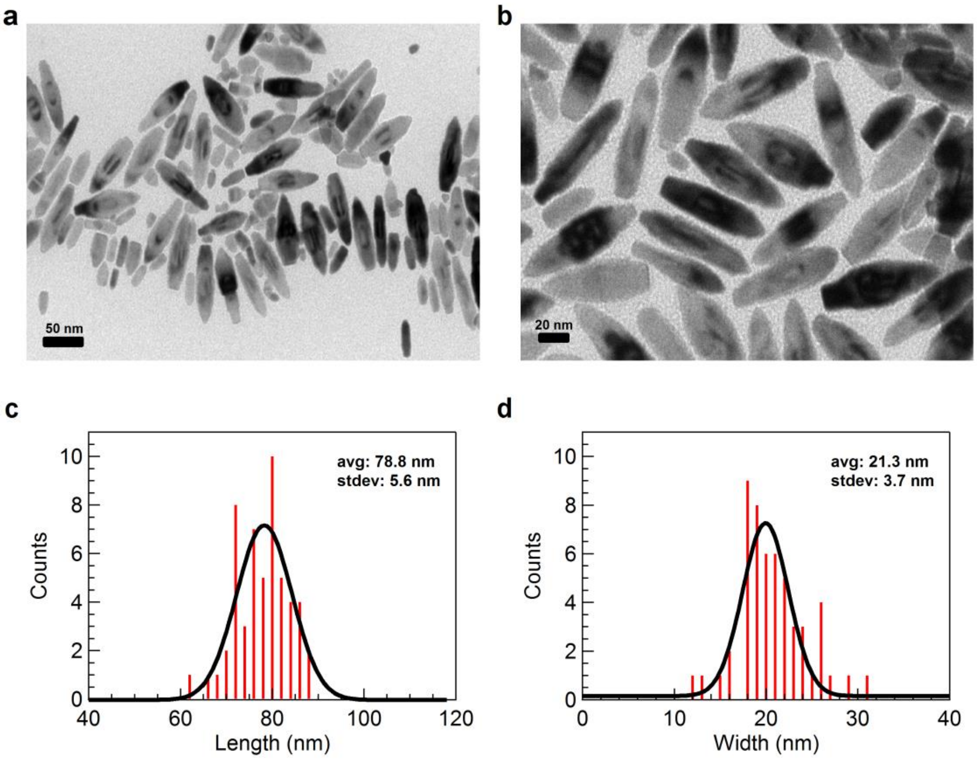

Supplementary Figure 2. CdSe/CdS RIR synthesis without the addition of $CdCl_2$. When performing the synthesis without the addition of $CdCl_2$, we typically produced smaller CdS nanocrystals as well, and the RIR nanocrystal width and length were slightly reduced (by 28% and 10% respectively). a) TEM image of the crude reaction mixture. b) TEM image of the sample after selective precipitation to remove the small CdS particles. c)-d) Histograms of the length and diameter distribution, obtained by analysis of 50 RIR heteroNCs.

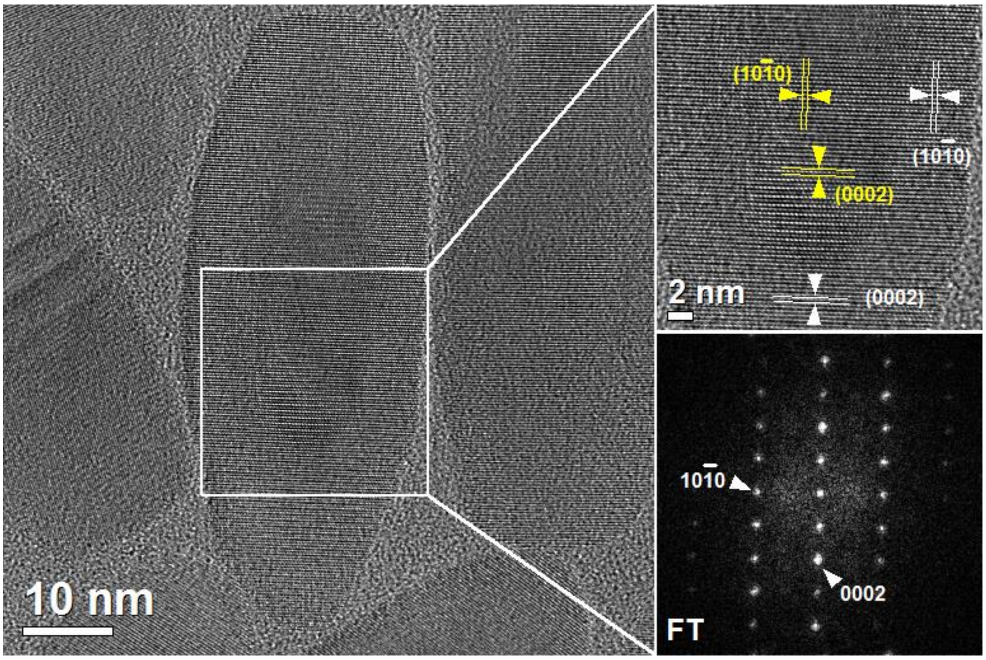

Supplementary Figure 3. HRTEM image of RIR Sample III, with zoom and corresponding FFT.

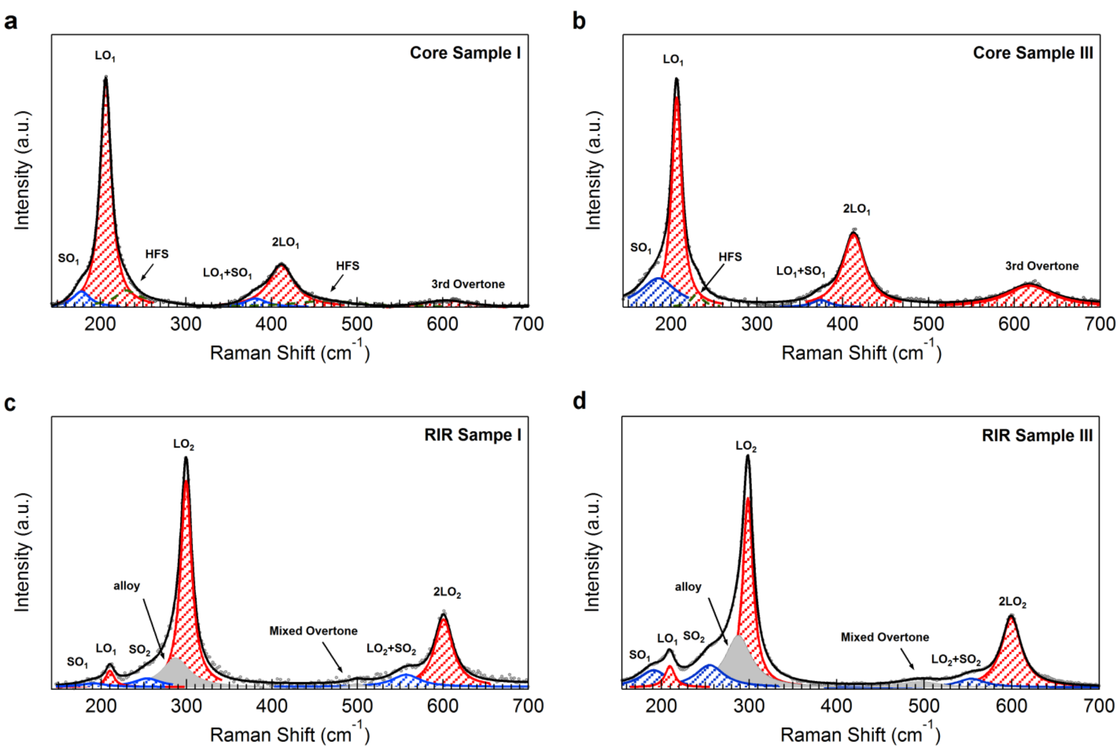

Supplementary Figure 4. Raman spectra with fit (black line), and individual phonon modes for the CdSe NCs sample I (a) and sample III (b). Corresponding CdSe/CdS RIR sample I and RIR sample III are shown in panels (c) and (d), respectively. Surface optical (SO) modes for CdSe (SO_1) and CdS (SO_2) are indicated in blue, longitudinal optical (LO) modes (LO_1: CdSe, LO_2: CdS) in red. Alloy modes are shown in grey, and the high-frequency shoulder (HFS) with a green line.

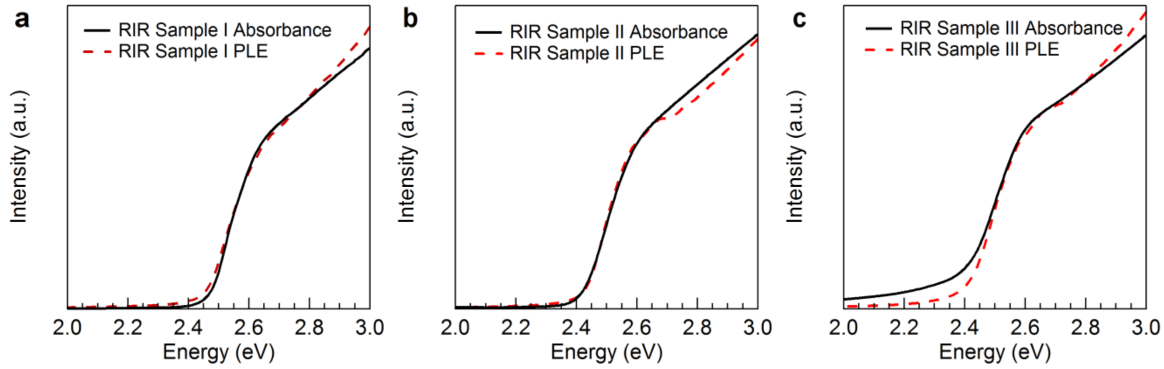

Supplementary Figure 5. PL excitation spectra for the three RIR NCs. In all cases, the PLE follows the absorbance spectrum. For RIR Sample III, minor deviations occur, most likely due to some Rayleigh scattering giving rise to a low energy tail in the absorbance spectrum.

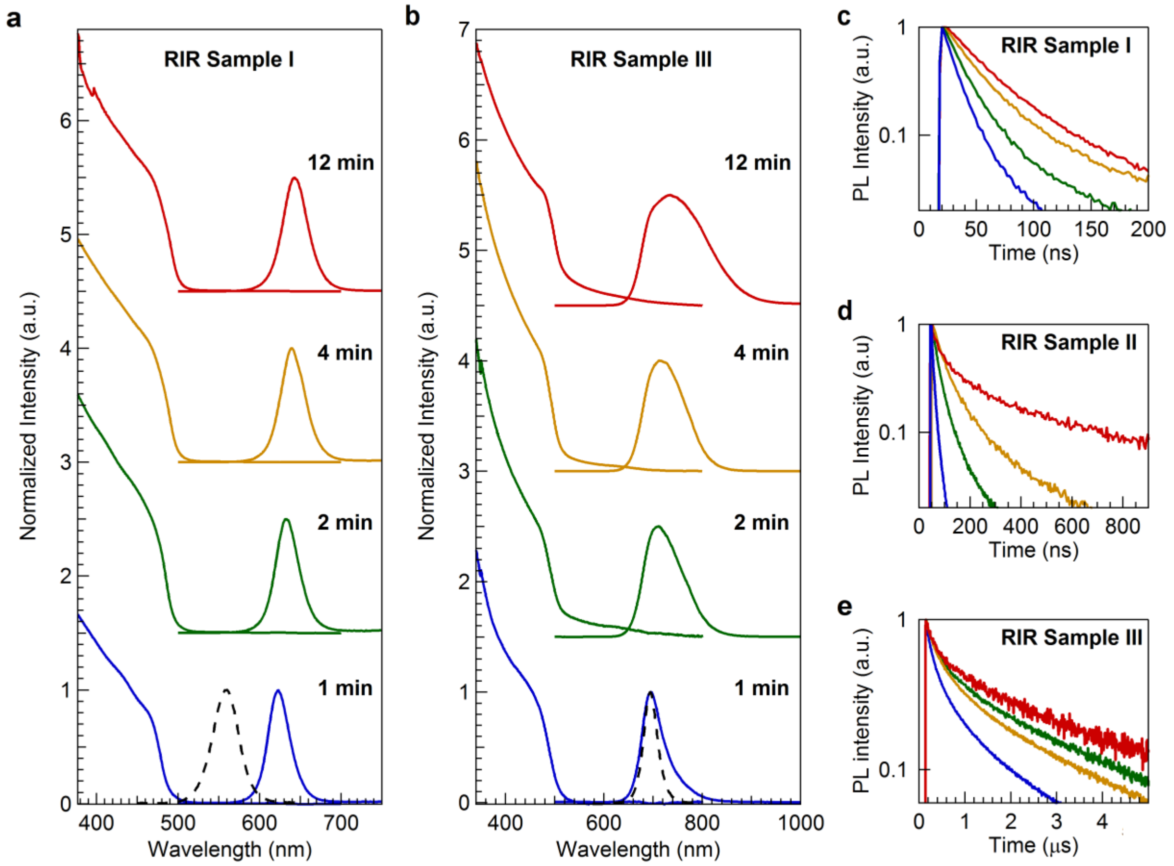

Supplementary Figure 6. Series of absorbance and PL spectra for RIR Sample I (panel a) and Sample III (panel b). Aliquots were taken during the growth of the different samples, and show a gradual red shift of the PL emission. The PL spectrum of the CdSe core nanorods is shown for comparison (dashed line). Corresponding time-resolved decay traces are shown in panels c-e, with a continuous increase in the PL lifetime during shell growth.

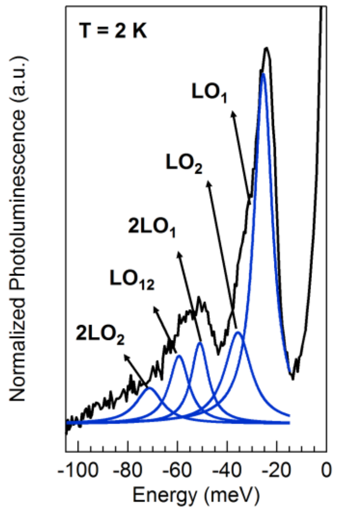

Supplementary Figure 7. The FLN spectrum of RIR sample I, excited at 2.07 eV, together with a fit with five Gaussian components. Peaks can be assigned to the longitudinal optical (LO) modes of CdSe (LO_1 = 25 meV) and CdS (LO_2 = 35 meV), with respective overtones at 50 meV and 70 meV. A mixed phonon overtone is observed at LO_{12}= 60 meV.[1]

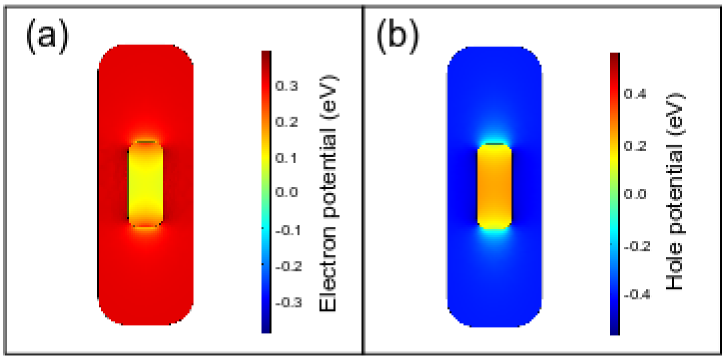

Supplementary Figure 8. RIR potential felt by the exciton electron (a) and hole (b) when piezoelectric fields are not considered. The calculation includes quantum confinement, strain and Coulomb attraction, but no piezoelectric field. For electrons the CdSe core potential is raised by ~0.1 eV due to hydrostatic compression (for holes it is lowered by ~0.2 eV), but the band alignment remains type I, favoring electron and hole localization inside the core. The potential is symmetric along the long axis of the RIR, which rules out the spatial separation of electrons and holes in this direction.

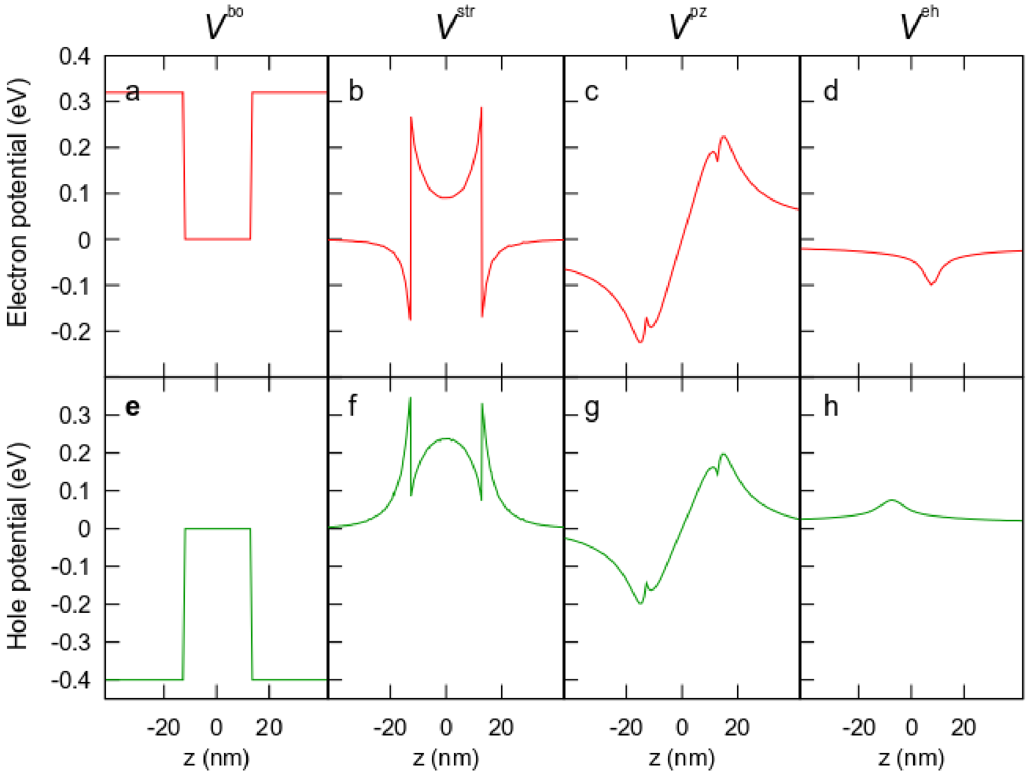

Supplementary Figure 9. Plot of the different components of the RIR conduction and valence band potential. (a-d): potential seen by the electron; (e-h) potential seen by the hole. From left to right: band-offset (V^{bo}), strain potential (V^{str}), piezoelectric field (V^{pz}), and Coulomb potential generated by the opposite carrier (V^{eh}). The strain potential raises the bottom of the conduction band, but for this RIR (sample III) the overall effect of (a) and (b) leads to a type I band offset. The piezoelectric potential, on the other hand, creates potential wells for electrons and holes on the opposite sides of the rod. Because the resulting system is type-II, the Coulomb interactions between the electron and the hole are fairly weak.

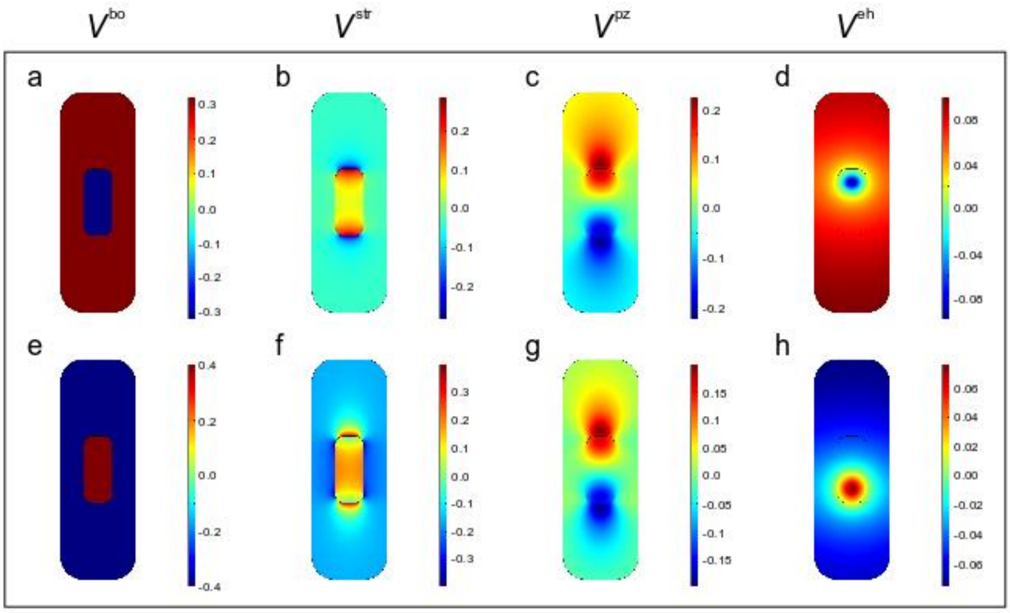

Supplementary Figure 10. Same as supplementary figure 9 but with a 2D view. All potentials are in eV.

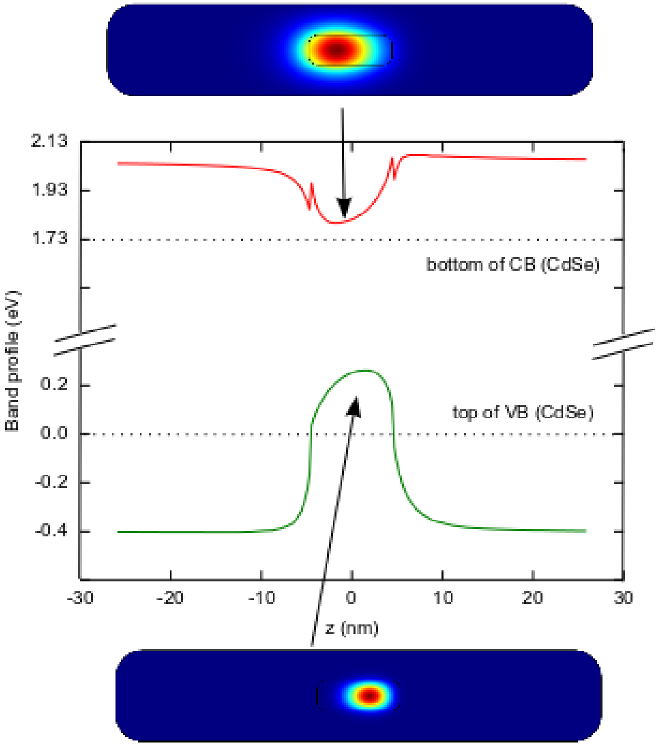

Supplementary Figure 11. Plot of the band structure and ground state wave functions for sample RIR I, and corresponding ground state exciton electron and hole wave functions. The piezoelectric field strength is comparable to the longitudinal confinement. This results in a more symmetric potential and wave functions closer to the center of the core compared to RIR Sample III (see also figure 4 in the main text). As a result, the electron-hole overlap is much larger (supplementary table 3).

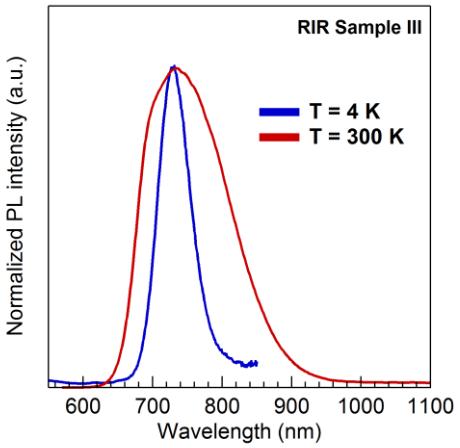

Supplementary Figure 12. Comparison of the PL spectrum of RIR sample III, measured at room temperature and 4 K, respectively. Multiexciton emission spectroscopy was performed at 4 K in order to improve the PL linewidth and enhance the visibility of any shifts in the PL spectrum.

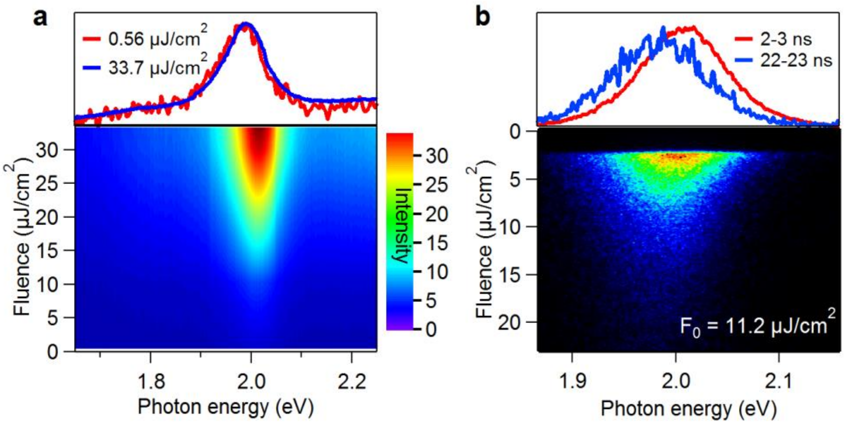

Supplementary Figure 13. a) The PL spectra of RIR Sample I under increasing fluence show only a slight rise of the PL on the blue side of the emission band, characteristic for biexciton emission in quasi-type II CdSe/CdS heteroNCs.[2] b) Time-resolved streak camera image collected at a fluence of 11.2 μJ.cm^{-2}. The spectrum after 22 ns is comparable to the single exciton emission spectrum (panel a), allowing to deduce a 28 meV blue shift for the MX emission.

Supplementary Table 1. Summary of the phonon frequencies extracted from fitting the Raman spectra with Lorentzian peaks. The full width at half maximum is reported between brackets. The line width of the peaks marked with * has been fixed to avoid excessive broadening; this did not significantly affect the reported peak position.

	Core Sample I (cm^{-1})	RIR Sample I (cm^{-1})	Core Sample III (cm^{-1})	RIR Sample III (cm^{-1})
**SO_1 (CdSe)**	178 (24)	191 (37)	185 (49)	191 (39)
**LO_1 (CdSe)**	206 (16)	210 (13)	206 (14)	209 (13)
**LO_1+SO_1**	381 (31)	-	374 (25)	-
**$2LO_1$**	411 (36)	-	413 (31)	-
**3rd Overtone**	603 (79)	-	617 (67)	-
**SO_2 (CdS)**	-	254 (38)	-	255 (39)
**$CdSe_xS_{1-x}$ alloy**	-	287 (39)	-	288 (37)
**LO_2 (CdS)**	-	299 (16)	-	299 (14)
**Mixed Overtone**	-	499 (23)	-	495 (59)
**LO_2+SO_2**	-	557 (40*)	-	554 (40*)
**$2LO_2$**	-	601 (25)	-	599 (27)

Supplementary Table 2. Size analysis of the core region as obtained via HAADF-STEM and EDS, and mean dilatation maps of the CdSe/CdS core/shell NCs. We used HAADF-STEM images of 40 RIRs, with the cores of CdSe clearly observable due to the difference in Z-contrast with respect to the CdS shells. Average dimensions of the cores were calculated from EDS by measuring the intensity of the Se Kα line. Line scans were performed along the two main axes of 10 RIRs. For the peak pair analysis (PPA) method of the strain maps, a Bragg-filtered image was obtained by selecting the main diffraction spots from the 2D FFT of the RIR. An unperturbed central area of the RIR was taken as reference for calculating the strain-modified lattice parameters of the whole crystal. The average length and width of those areas were calculated from the mean dilatation maps using 10 NCs. The error on the average values includes both the measurement error and the dispersion on the CdSe core sizes.

	EDS Line scan	HAADF-STEM	Strain map
**Average length (nm)**	21.8 ± 2.1	22.1 ± 2.0	32.0 ± 2.1
**Average width (nm)**	10.6 ± 1.0	9.9 ± 1.02	11.6 ± 0.7

Supplementary Table 3. Comparison of calculated and experimental excitonic properties for the different CdSe/CdS NCs under study.

					**CBO=0.32 eV/0.12 eV**		
**Sample**	**Diameter (nm)**	**Length (nm)**	**Exp. band gap emission (eV)**	**Exp. Lifetime (ns)**	**Calc. Electron-hole Overlap**	**Calc. band gap emission (eV)**	**Calc. Lifetime (ns)**
**Core Sample I**	3.2	8.9	2.22	14	0.923	2.22	14
**Core Sample II**	4.8	15.0	1.96	15	0.918	1.97	16
**Core Sample III**	10.8	25.6	1.79	24	0.922	1.82	17
**RIR Sample I**	9.7	52.0	1.93	102	0.764 / 0.55	1.81 / 1.74	25 / 50
**RIR Sample II**	9.8	43.8	1.85	853	0.684 / 0.13	1.69 / 1.68	34 / 839
**RIR Sample III**	28.9	84.8	1.69	4450	$6.6\cdot10^{-3}$ / $2.7\cdot10^{-6}$	1.59 / 1.47	$3.9\cdot10^{5}$ / $2.5\cdot10^{12}$

Wave function overlaps are calculated as $S_{eh} = \langle \Psi_e | \Psi_h \rangle$, where Ψ_e and Ψ_h are the ground state exciton electron and hole wave functions obtained from the self-consistent Schrödinger-Poisson iteration. The band gap emission is the ground state exciton energy E_X. We have assumed that the conduction and valence bands are split by E_{gap} =1.88 eV, which is slightly larger than the optical gap (1.74 eV) to exclude the exciton binding energy, as it is already implicit in the self-consistent electron and hole energies. For the lifetime calculation we consider that $1/\tau = k * E_X * S_{eh}^2$, where k is a constant fitted to match the experimental lifetime τ of Core Sample I.

One can see that increasing the size of core-only NCs has a weak effect on the radiative lifetimes. Only the addition of the CdS shell gives rise to a sizable decrease of the electron-hole overlap, resulting in longer lifetimes. This behavior is particularly pronounced for large RIRs owing to the weaker longitudinal confinement. In large RIRs, the emission energy becomes smaller than the optical band gap of CdSe (1.74 eV). Two values of the conduction band offset (cbo) are used in the calculations to take into account the range of conduction band offsets given in literature.[3] In both cases, the predicted trends are qualitatively consistent with experiments, supporting the fact that piezoelectricity plays a central role in determining the optical properties of RIRs. This is further confirmed by the calculated τ when the piezo-electric effect is not considered: for a cbo of 0.32 eV (0.12 eV), we find 23 ns (39 ns), 22 ns (28ns) and 20 ns (25 ns), for RIR Sample I, II and III respectively.

Supplementary Table 4. Material parameters used in the k·p calculations. m_0 is the free electron mass, a relative dielectric constant of 3 is taken outside the RIR to account for the dielectric environment.

Description	Symbol	CdSe value	CdS value	Units	Ref.
Conduction band offset	cbo	0.0	0.32	eV	[4]
Valence band offset	vbo	0.0	-0.4	eV	[4]
Conduction band deformation potential xy	a_{xy}^{c}	-7.8	-8.2	eV	[5]
Conduction band deformation potential z	a_{z}^{c}	-1.76	-4.5	eV	[5]
Valence band deformation potential xy	a_{xy}^{v}	-1.34	-1.84	eV	(*)
Valence band deformation potential z	a_{z}^{v}	-7.8	-10.7	eV	(*)
Electron effective mass	m^{*}_{e}	0.11	0.21	m_0	[6]
Hole effective mass (in-plane)	$m^{*}{}_{h}^{xy}$	-0.483	-0.376	m_0	[7]
Hole effective mass (c axis)	$m^{*}{}_{h}^{z}$	-1.19	-0.746	m_0	[7]
Elastic modulus tensor	C_{xxxx}	7.41	8.65	$\cdot 10^{10}$ Pa	[8]
Elastic modulus tensor	C_{xxyy}	4.52	5.40	$\cdot 10^{10}$ Pa	[8]
Elastic modulus tensor	C_{xxzz}	3.89	4.73	$\cdot 10^{10}$ Pa	[8]
Elastic modulus tensor	C_{zzzz}	8.43	9.44	$\cdot 10^{10}$ Pa	[8]
Elastic modulus tensor	C_{xzxz}	1.34	1.50	$\cdot 10^{10}$ Pa	[8]
Elastic modulus tensor	C_{xyxy}	1.45	1.63	$\cdot 10^{10}$ Pa	[8]
Lattice constant (in-plane)	a	4.30	4.135	Å	[6]
Lattice constant (c axis)	c	7.01	6.749	Å	[6]
Density	d	5810	4820	$kg.m^{-3}$	[6]
Relative dielectric constant (in-plane)	ε_r^{xy}	9.29	8.28	-	[6]
Relative dielectric constant (c-axis)	ε_r^{z}	10.16	8.73	-	[6]
Piezoelectric constant	e_{31}	-0.16	-0.36	$C.m^{-2}$	[9]
Piezoelectric constant	e_{33}	0.347	0.16	$C.m^{-2}$	[9]
Piezoelectric constant	e_{15}	-0.138	-0.12	$C.m^{-2}$	[9]

(*) wurtzite valence band deformation parameters are usually given for multiband Hamiltonians with coupled heavy and light hole subbands (see e.g. Jeon et al.[10]). Because we

work with a single-band (decoupled) heavy hole Hamiltonian, we estimate deformation potential values which implicitly account for the coupling to near and remote bands. The diagonal kinetic energy and strain terms of heavy holes in the multiband Hamiltonian read:[11]

$$H_k = \frac{1}{2}\left(\nabla_x + \nabla_y\right)(L_1 + M_1)\left(\nabla_x + \nabla_y\right) + \frac{1}{2}\,\nabla_z\, M_2\, \nabla_z$$

$$H_s = \frac{1}{2}\,(l_1 + m_1)\left(\varepsilon_{xx} + \varepsilon_{yy}\right) + \; m_2\, \varepsilon_{zz}$$

And the corresponding effective single-band Hamiltonians:

$$H_k^{hh} = \frac{1}{2}\left(\nabla_x + \nabla_y\right)\frac{1}{m_h^{*xy}}\left(\nabla_x + \nabla_y\right) + \frac{1}{2}\,\frac{1}{m_h^{*z}}\,\nabla_z$$

$$H_s^{hh} = \frac{1}{2}\,a_v^{xy}\left(\varepsilon_{xx} + \varepsilon_{yy}\right) + \; a_v^{xy}\,\varepsilon_{zz}$$

Assuming the inclusion of non-heavy-hole bands scales the deformation potential parameters to the same extent as the mass parameters, one obtains:

$$a_v^{xy} = \frac{l_1 + m_1}{L_1 + M_1}\,\frac{1}{m_h^{*xy}}$$

$$a_v^{z} = \frac{m_2}{M_2}\,\frac{1}{m_h^{*z}}$$

L_1, M_1, M_2, l_1, m_1 and m_2 are inferred from Jeon et al.,[10] while $m^*_h{}^{xy}$ and $m^*_h{}^{z}$ are obtained from Laheld and Einevoll.[7]

Supplementary Note 1. Details on the epitaxial relationships.

The interface between CdSe cores (space group $P6_3mc$, JCPDS card 77-2307) and CdS shells (space group $P6_3mc$, JCPDS card 80-0006) of CdSe/CdS RIRs was analyzed in order to evaluate the epitaxial growth. The interface was aligned according to the following relationships (supplementary figure 3):

$$CdSe\ [0002]\ //\ CdS\ [0002]$$
$$CdSe\ [10\bar{1}0]\ //\ CdS\ [10\bar{1}0]$$

These two terms represent the (parallel) lattice alignment along the length and the width of the RIRs, respectively. The lattice mismatch parameter m between average lattice spacings of the two phases along a given direction is calculated from the average lattice spacing of phase 1 and 2, d_1 and d_2, respectively: $m = 2 \times |d_1 - d_2| / (d_1+d_2)$. In well oriented RIRs, the Fourier analysis of the HRTEM images allows to accurately calculate m by means of a filtered 2D-Fast Fourier Transform (FFT) of the image. We obtained m = 0.9% and 2.9% for the [0002] and $[10\bar{1}0]$ directions, respectively. Clearly, the average lattice mismatch obtained from the HRTEM image falls well below the unstrained mismatch of 3.9% and 4%, giving a first indication of the role of strain in the RIRs.

Supplementary Note 2. Raman spectroscopy on CdSe core and CdSe/CdS core/shell NCs.

For CdSe nanorods (supplementary figure 4, panels a and b), the Raman spectra are in agreement with literature data[12,13] (see also supplementary table 1), with the observation of fundamental surface optical (SO_1, blue peaks) and longitudinal optical (LO_1, red peaks) phonon modes and their overtones. We also detected small high frequency shoulders (HFS, green lines), at 230 cm^{-1} and 460 cm^{-1} (ref 12). For CdSe/CdS RIR NCs (panels c and d), we again find the SO and LO modes, now for both CdSe (SO_1, LO_1) and CdS (SO_2, LO_2). The latter are more intense due to the larger volume of the CdS shell. The CdSe phonon modes are shifted to higher frequencies confirming the crystal lattice compression.[12,13] However, accurate deconvolution of the peaks also required the addition of modes arising around 290 cm^{-1} and 500 cm^{-1} (grey peaks), that can be attributed to lattice vibrations in a $CdSe_xS_{1-x}$ alloy region at the core/shell interface.[12]

Supplementary Note 3. Synthesis details for size-controlled CdSe nanorods.

For 4.8 nm x 15.0 nm cores, we degassed and heated 3 g of TOPO, 25 mg of CdO, 40 mg of HPA and 180 mg of ODPA to 340 °C, when 1 mL of a 0.08 M TOPSe solution was added. The nanorods where grown for 1 min. The smallest, 3.8 nm x 8.9 nm cores, were synthesized starting from 3 g of TOPO, 25 mg of CdO, 40 mg of HPA, 180 mg of ODPA, injecting 0.5 mL of a 0.08 M TOPSe solution at 320 ^{o}C and growing the nanorods for 20 sec. At the end of each reaction the solution was rapidly cooled and 10 mL of toluene was added at 90 °C. The particles were purified by adding 5 mL of ethanol and centrifuging them at 3000 rpm for 5 min, after which the nanorods were dispersed in toluene. This process was repeated 3 times, with final dispersion in 3 mL of toluene. The CdSe nanorod concentration was determined by combining the size, obtained via TEM, with elemental analysis using inductively-coupled plasma optical emission spectroscopy. The samples were finally dried in a glove box and dispersed in TOP at a concentration of 3 µM.

Supplementary References

1. García-Santamaría, F. *et al.* Breakdown of Volume Scaling in Auger Recombination in CdSe/CdS Heteronanocrystals: The Role of the Core−Shell Interface. *Nano Lett.* **11,** 687–693 (2011).
2. Sitt, A., Della Sala, F., Menagen, G. & Banin, U. Multiexciton Engineering in Seeded Core/Shell Nanorods: Transfer from Type-I to Quasi-type-II Regimes. *Nano Lett.* **9,** 3470–3476 (2009).
3. Steiner, D. *et al.* Determination of Band Offsets in Heterostructured Colloidal Nanorods Using Scanning Tunneling Spectroscopy. *Nano Lett.* **8,** 2954–2958 (2008).
4. Wei, S.-H., Zhang, S. B. & Zunger, A. First-principles calculation of band offsets, optical bowings, and defects in CdS, CdSe, CdTe, and their alloys. *J. Appl. Phys.* **87,** 1304–1311 (2000).
5. Park, S.-H. & Cho, Y.-H. Strain and piezoelectric potential effects on optical properties in CdSe/CdS core/shell quantum dots. *Journal of Applied Physics* **109,** 113103 (2011).
6. Madelung, O. *Semiconductors: Data Handbook*. (Springer, 2004).
7. Laheld, U. E. H. & Einevoll, G. T. Excitons in CdSe quantum dots. *Phys. Rev. B* **55,** 5184–5204 (1997).
8. Sadao, A. *Handbook of Physical Properties of Semiconductors*. (Kluwert Academic Group, 2004).
9. Madelung, O., Rössler, U. & Schulz, M. *II-VI and I-VII Compounds; Semimagnetic Compounds*. **Volume 41B,** (Springer Materials, 1999).
10. Jeon, J.-B., Sirenko, Y. M., Kim, K. W., Littlejohn, M. A. & Stroscio, M. A. Valence band parameters of wurtzite materials. *Solid State Communications* **99,** 423–426 (1996).
11. Voon, L. C. L. Y. & Willatzen, M. *The k•p Method: Electronic properties of semiconductors*. (Springer, 2009).
12. Dzhagan, V. M. *et al.* Raman- and IR-Active Phonons in CdSe/CdS Core/Shell Nanocrystals in the Presence of Interface Alloying and Strain. *J. Phys. Chem. C* **117,** 18225–18233 (2013).
13. Tschirner, N. *et al.* Interfacial Alloying in CdSe/CdS Heteronanocrystals: A Raman Spectroscopy Analysis. *Chem. Mater.* **24,** 311–318 (2012).
